# Introduction to Pharmaceutical Co-amorphous Systems Using a Green Co-milling Technique

**DOI:** 10.1021/acs.jchemed.3c00036

**Published:** 2023-03-23

**Authors:** Joana F. C. Silva, Mário T. S. Rosado, Teresa M. R. Maria, Pedro S. Pereira Silva, Manuela Ramos Silva, M. Ermelinda S. Eusébio

**Affiliations:** † CQC-IMS, Dep. de Química, Universidade de Coimbra, Rua Larga, Coimbra 3004-535, Portugal; ‡ CFisUC, Dep. de Física, Universidade de Coimbra, Rua Larga, Coimbra 3000-370, Portugal

**Keywords:** Graduate Education/Research, Hands-On Learning/Manipulatives, Mechanochemistry, Physical Chemistry, Drugs/Pharmaceuticals, Green Chemistry, Phases/Phase Transitions, Solid State Chemistry

## Abstract

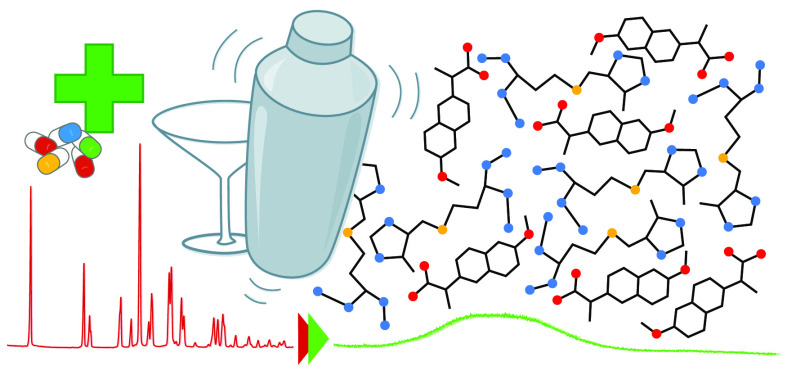

The concept of co-amorphous systems is introduced in an integrated laboratory experiment, designed for advanced chemistry students, using solvent-free, environmentally friendly mechanochemistry. The dual-drug naproxen-cimetidine co-amorphous system (NPX-CIM) is investigated as an example of the emergent field of medicinal mechanochemistry. Students are trained in solid-state characterization techniques including X-ray powder diffraction, Fourier-transform infrared spectroscopy, and thermal analysis by differential scanning calorimetry. This lab experiment also provides an opportunity to discuss the relevance of different solid forms of pharmaceutics, emphasizing particular properties of disordered materials. This experiment can easily fit the curriculum of any Chemistry or Pharmacy master level degree in courses dealing with instrumental analysis, solid state chemistry, or green chemistry, for classes of 6 to 18 students, in a 5-h lab session. Suggestions to adapt it to the use of a single characterization technique are provided.

## Introduction

Many active pharmaceutical ingredients (API) do not have the desired physicochemical properties to be directly suitable for formulation development. One of the greatest challenges faced by the pharmaceutical industry results from the increasing number of poorly water-soluble drugs. Poor aqueous solubility is a critical factor that limits the development of oral pharmaceutical solids and of new chemical entities.^1^ It is a recognized fact that the majority of drug candidates in pharmaceutical development exhibit poor water solubility and are categorized as Class II or Class IV according to the Biopharmaceuticals Classification System (BCS).^2,3^ Addressing this issue, the pharmaceutical industry has developed several strategies that include searching for appropriate solid forms such as polymorphs, salts, co-crystals, and amorphous phases.^4^ Using the amorphous form of poorly water-soluble drugs has become one of the most effective approaches to improve their solubility and dissolution rate and thus enhance drug bioavailability.^5−7^

Compared to their crystalline counterparts, amorphous solids lack the long-range order of molecular packing and have higher internal energy, making them prone to crystallization. An emergent approach for stabilizing the amorphous phase is forming co-amorphous systems. Pharmaceutical co-amorphous systems are homogeneous amorphous mixtures made of an API and one or more low-molecular-weight excipients and/or other drug molecules.^3,8−11^ The intermolecular interactions between the drug and the co-former and/or the effect of mixing are responsible for an increase of stability of the amorphous phases, avoiding crystallization. When compared to other amorphous dispersions combining drug with polymers, co-amorphous systems show the greater advantage of allowing a higher API load.

Nowadays, mechanochemistry, the science and technology using mechanical activation to achieve chemical transformations, has flourished as a green method widely investigated as an alternative to traditional chemical procedures. Mechanochemistry was considered one of the ten chemical innovations that will change our world, according to IUPAC in 2019.^12,13^ Its advantages result from the solvent-free nature of most mechanochemical protocols, reducing waste production.^14,15^ Since neither organic solvents nor high temperatures are involved, mechanochemistry has become an essential subject of interest in pharmaceutical sciences and a green, high yield approach for disordered pharmaceutical materials synthesis.^16−18^ Due to the ease of handling, mechanochemical methods, like ball milling, are the most widely used techniques, having demonstrated their potential to generate stable co-amorphous systems.^5,11^ Advantages of milling include low chemical degradation and high recovery compared to other preparation methods, like quench cooling, involving high energy consumption, and solvent evaporation, adding the use of bulk organic solvents to the energy inefficiency.

**Figure 1:**
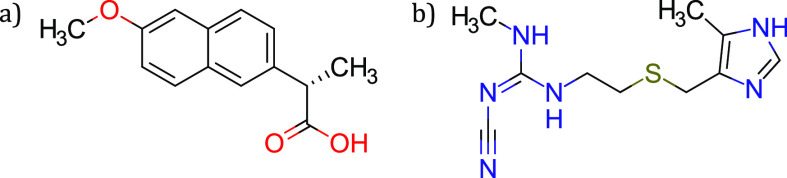
Chemical structures of (a) naproxen (NPX) and (b) cimetidine (CIM).

Herein we report a laboratory experiment aiming at the mechanochemical synthesis and the characterization of the dual-drug (1:1) co-amorphous system made up of (*S*)-naproxen and cimetidine, see Figure 1.^19,20^ Naproxen (NPX) is a BCS class II nonsteroidal anti-inflammatory drug frequently sold as the (*S*)-enantiomer. Its side effects include gastrointestinal disorders. On the other hand, cimetidine (CIM) is used to treat heartburn and peptic ulcer and has outstanding importance in the pharmaceutical industry, being the first drug to reach $1 billion in annual sales.^21^ The combination of the two API in the same phase can be synergetic because cimetidine can ameliorate naproxen side effects. In addition, a dual-drug strategy treatment has the recognized advantage of a higher patient compliance.^22,23^

The formation of the co-amorphous systems is achieved at room temperature by neat grinding, and the sample is characterized by X-ray powder diffraction (XRPD), differential scanning calorimetry (DSC), and Fourier-transform infrared spectroscopy (FTIR). The same experimental techniques are used to characterize the starting pure materials before and after being submitted to the same mechanochemical procedure, verifying eventual solid phase changes induced by grinding. Finally, the results of the three procedures are compared and discussed.

While the solid phase characterization used in this laboratory experiment was intended as a demonstration of the complementary use of physical chemistry instrumental analysis techniques, this work may be adapted for actual experimental work with only one of them due to limited instrument availability or shorter lab session duration. Hence, each instructor may adopt one individual characterization technique, exploring its particular capabilities. A few examples can be found in the instructor notes provided as Supporting Information.

In this project, the students are introduced to the concept of co-amorphous systems, contextualized in the field of the diversity of solid forms of active pharmaceutical ingredients. It is also an opportunity to discuss thermodynamic and kinetically stable phases. Additionally, the importance of using more sustainable solvent-free methodologies such as mechanochemistry is emphasized by comparison with other amorphization procedures. Another recent lab experiment was published that used XRPD simulations to identify naproxen enantiomers by Rietveld refinement.^24^

This lab session also contributes to consolidate knowledge on XRPD, FTIR, and DSC physicochemical characterization techniques and to understand their application in the analysis of organic solid-state materials. The pedagogical goals of the experiment and the associated discussion are summarized in Table 1.

**Table 1:** Pedagogical Goals of the Laboratory Experiment and Associated Discussion

Topics Focused and Methods Studied	Learning Outcomes
Co-amorphous systems: fundamentals and applications	Students will understand what co-amorphous systems are, how to obtain them, and their relevant properties and applications in the pharmaceutical field.
	They will be able to identify co-amorphous systems in the context of the diversity of solid forms of API.
Thermodynamic and kinetic stability	Students will be able to distinguish the concepts of thermodynamic and kinetically stable phases.
Introducing to mechanochemistry	Students will recognize the advantages of the co-milling process as a green approach to supramolecular synthesis. Mechanochemistry can satisfy more than one of the green chemistry principles in a single experimental process. See Table S1 in the Supporting Information.
Basic introduction to polymorphism	Students will recognize that solid materials can undergo a change in their crystal structure as a result of physical processing, such as milling, which will lead to changes in their physicochemical properties.
X-ray powder diffraction	Students will be able to discriminate between different crystalline forms based on the characteristic diffraction patterns. They will also be able to identify an amorphous solid by the absence of peaks and the presence of a halo in the diffractogram.
FTIR-ATR spectroscopy	Students will assign the most important bands in the IR spectra of the product and starting materials to their functional groups, recognize changes due to amorphization, and look for possible changes in the spectra that may be due to specific intermolecular interactions in the co-amorphous systems.
Thermal analyses by DSC	Students will be capable of determining the glass transition temperature of amorphous solids and melting points of crystalline materials.

During the experiment, some guiding questions can be asked to promote discussion between the instructor and students and may help to introduce each technique and fundamental concepts previously mentioned. Guiding questions are provided in the Supporting Information (see the Instructor Notes).

This experiment was designed for advanced graduate students attending the first year of a Master in Chemistry degree and was performed in a 5-h lab session. It can easily fit the curriculum of any Chemistry or Pharmacy master level degree in courses dealing with instrumental analysis, solid state chemistry, or green chemistry. Prior to experiments, students were asked to read general information about co-amorphous systems and the techniques to be used.^6,8,17^ In a 60-min prelab session, the instructor contextualized co-amorphous systems and the project in the field of solid forms of API in an interactive discussion with student participation. The fundamentals of mechanochemistry and its advantages as a green methodology as well the basics of the characterization techniques were also discussed. After this introductory session, the class was divided into three groups, where the students worked in pairs. For students that did not have previous contact with the characterization techniques, these can be the subject of a previous session dealing with their fundamentals and experimental details. Planning of the lab session, detailed experimental conditions, safety precautions, hazards, and experimental are also provided in the Supporting Information (see the Instructor Notes).

Students’ assessment was performed on the basis of the instructor follow-up of their commitment during the prelab and lab sessions, based on the evaluation of their lab reports, and the answers to the questions listed in the Supporting Information (see the Instructor Notes). The discussion of the results and the evaluation of the answers to these questions, included in the end of the lab reports, allowed the assessment of the specific pedagogical goals listed in Table 1.

## Experimental Section

### Mechanochemistry

Formation of co-amorphous systems was performed by neat co-milling at room temperature. The amorphization of a total mass of ≈100 mg of an equimolar mixture of crystalline naproxen (NPX) and cimetidine (CIM) was mechanically activated using a mixer mill with 10 mL stainless steel jars and two balls (ø = 7 mm) for 60 min at 30 Hz. Pure crystalline compounds were submitted to the same milling process.

### Characterization

All samples were characterized using DSC, XRPD, and FTIR.

X-ray powder diffraction was used to characterize the starting materials and the solid products of the milling processes. The absence of long-range lattice periodicity and crystallographic planes could be confirmed from the observation of the typical halos of disordered materials^25^ and the absence of Bragg peaks, confirming the success of amorphization by the grinding process.

The infrared spectra in the mid-IR region give information on the intramolecular vibrational modes, which can be used to investigate molecular structure and give evidence of the structural differences among different solid forms. Thus, the starting materials and milled samples were also characterized by FTIR. The most characteristic functional groups of each API, giving rise to specific bands in the spectra, were identified. FTIR spectral features are also sensitive to intermolecular interactions and can give insight into changes in hydrogen bond interactions between API molecules and the formation of new interactions between the API and the co-former in co-amorphous systems, which are related to their stability.^26,27^

Thermal analysis is an indispensable and well-established tool for the characterization of co-amorphous systems. DSC measurements were used to confirm a single glass transition event, as expected for a co-amorphous mixture, and to determine the glass transition temperature (*T*_g_) of the amorphous phases. *T*_g_ were determined from the characteristic step change in heat capacity observed in the thermogram and calculated as the midpoint between the onset and endset temperatures of this event.^28^

XRPD patterns, FTIR spectra, and DSC data obtained for the co-amorphous NPX-CIM system were compared with those of the starting and milled pure materials.

Representative student XRPD and FTIR spectra and DSC thermogram are provided as Supporting Information.

## Hazards

The entire experiment must be conducted under appropriate safety practices. Students should wear gloves, safety goggles, and lab coats, avoiding contact of the reagents with the skin. Naproxen and cimetidine powders are inhalation hazards. In addition, all the experiments must be carried out under the instructor’s supervision. Detailed data about all the chemicals used throughout the experiment are given in the Supporting Information.

## Results and Discussion

**Figure 2:**
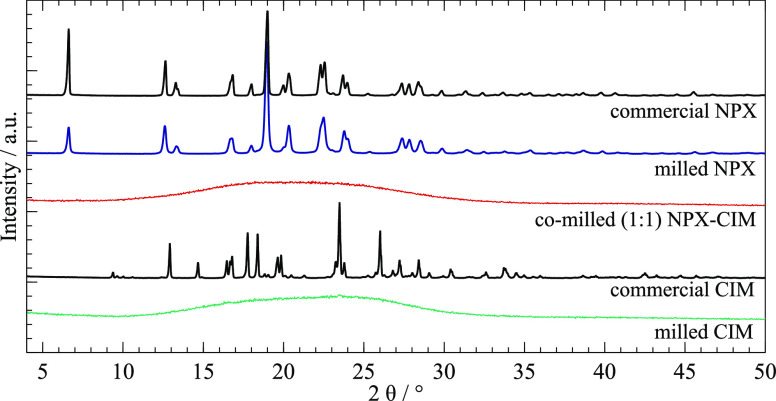
X-ray powder diffractograms of commercial and milled NPX and CIM, and co-milled (1:1) NPX-CIM.

Figure 2 shows the XRPD diffraction patterns of pure NPX and CIM as the starting crystalline materials and after milled separately, and of the co-milled NPX-CIM mixture. Characteristic peaks of the crystalline samples are described in detail in the Supporting Information. The students must compare these patterns to those simulated for reported crystalline structures of pure NPX and pure CIM found in the Cambridge Structural Database (CSD), where we could identify our starting sample of commercial crystalline NPX as the form described by Kim, Song, and Park^29^ (see Supporting Information, Instructor Notes). The milled sample of NPX remained crystalline and in the same solid form. These results also provide an opportunity for the instructor to discuss with the students the peak broadening effect that results from particle size reduction in the milling process. On the other hand, starting crystalline CIM was identified as the polymorph described by Cernik et al.^30^ (see Supporting Information, Instructor Notes). However, after milling, CIM diffraction pattern showed a typical halo, indicating the formation of an amorphous phase.

The instructor may call attention to the different outcomes obtained from the same mechanochemistry procedures to introduce the discussion about some factors that affect amorphization by grinding: typically, larger, irregularly shaped, more conformationally flexible molecules are more prone to amorphization rather than crystallization. Smaller, stiffer molecules like naproxen (when compared with cimetidine) are more likely to acquire long-range order and pack into crystals.

The XRPD data for co-milled NPX-CIM mixtures at a 1:1 molar ratio lack any Bragg peaks, also showing only the typical halo of amorphous phases. The milling procedure resulted in an amorphous phase for the NPX-CIM mixture because the more flexible CIM molecules effectively create new intermolecular interactions with the NPX molecules. These interactions segregate the NPX molecules, preventing their mutual association as a crystalline phase and, hence, stabilizing the NPX-CIM mixture as a single co-amorphous system. This shows that CIM has the relevant properties to act as an amorphization co-former for NPX.

Besides allowing discussion on the importance of XRPD in the characterization of solid-state materials, these experiments are also an opportunity to concisely introduce the basics of polymorphism and the effects that sample processing can have in the solid-state form of API, as observed for cimetidine.^31−33^ Despite not being the focus of this laboratory experiment, students should be made aware that it is well-known that processes like mechanochemistry, melt crystallization, and compression can lead to the formation of different polymorphs (different crystalline structures of the same substance), which can exhibit different physicochemical properties (e.g., melting temperature, solubility, dissolution rate, etc.).

Vibrational spectroscopy techniques are especially appropriate to characterize molecular interactions in solid phases. It is commonly known that peak broadening and peak shifts can occur when comparing the crystalline and the amorphous form of solids due to altered molecular arrangement and short-range order in the amorphous phase. ATR-FTIR spectra were analyzed to observe the effects of amorphization in the general profile of the vibrational bands, and the effects of intermolecular association between the components of the mixture.

**Figure 3:**
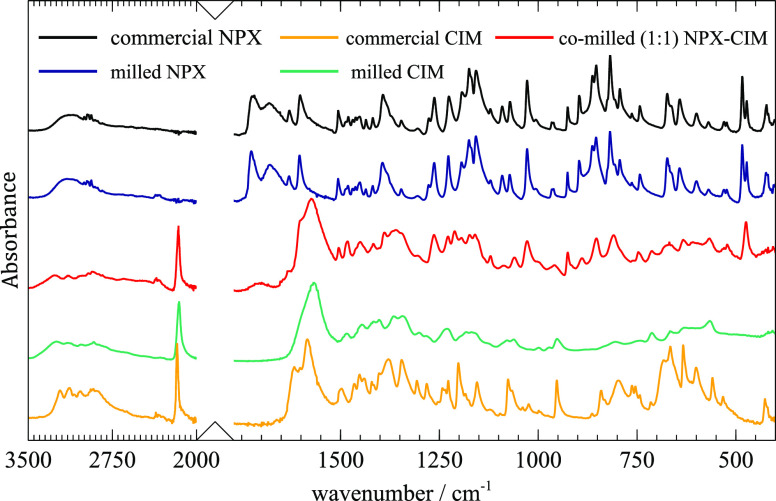
FTIR spectra of commercial and milled NPX, commercial and milled CIM, and co-milled (1:1) NPX-CIM.

Figure 3 shows the FTIR spectra of commercial and milled NPX, milled CIM, commercial CIM, and co-milled NPX-CIM. As expected, the spectrum of milled NPX is very similar to that of commercial sample since milling did not change the solid form.

On the other hand, there is a general band broadening in the FTIR spectrum of the amorphous phase of CIM obtained upon milling, as expected. In particular, the peaks at 3220, 3133, and 3036 cm^–1^ assigned to the NH stretching modes in the crystalline phase keep a similar although broader profile after amorphization, while the prominent ν(C≡N) band shifts from 2173 to 2155 cm^–1^. The fingerprint region of the spectrum of amorphous CIM shows several noticeable differences, which can indicate a different local molecular arrangement, namely in the features at 1616, 1378, 1202, 1077, 799, and 700–600 cm^–1^ (see the Supporting Information, Instructor Notes).

If there are not significant new intermolecular interactions between the components of a mixture, its vibrational spectrum is expected to be mostly a sum of the spectra of the starting materials. In general, other changes in the spectra of mixtures indicate the formation of different intermolecular interactions between the components. The IR spectrum of the co-amorphous mixture shows notable differences compared to the spectra of starting materials. The ν(C≡N) band, for instance, appears at a different wavenumber (2160 cm^–1^) (see the Supporting Information, Instructor Notes). Previously published results suggest that there is salt formation in the NPX-CIM amorphous mixture.^34−36^ This is supported by our IR spectrum, which shows the absence of the strong peak assigned to the carbonyl stretching mode in the carboxylic acid group (≈ 1728 cm^–1^). The bands corresponding to the carboxylate product are difficult to assign due to overlapping with others. Nevertheless, several NPX bands are identifiable, despite the expected general broadening, for instance in the peaks appearing at 1029 and 475 cm^–1^.

Calorimetric experiments are essential to introduce and explain the relevant properties that characterize an amorphous material. One of the most important is the glass transition temperature, *T*_g_, when, upon heating, the amorphous solid acquires significant molecular motion. The inverse process, vitrification, can be observed upon cooling. Below this characteristic temperature, there is a solid glassy phase, with restricted molecular motions, while above there is a supercooled liquid. Both of these states are not at thermodynamical equilibrium and lack long-range order. The identifying characteristic of a glass transition in a DSC thermogram is a step in the baseline of the heating curve, related to the heat capacity change between the glass and the supercooled liquid.

**Figure 4:**
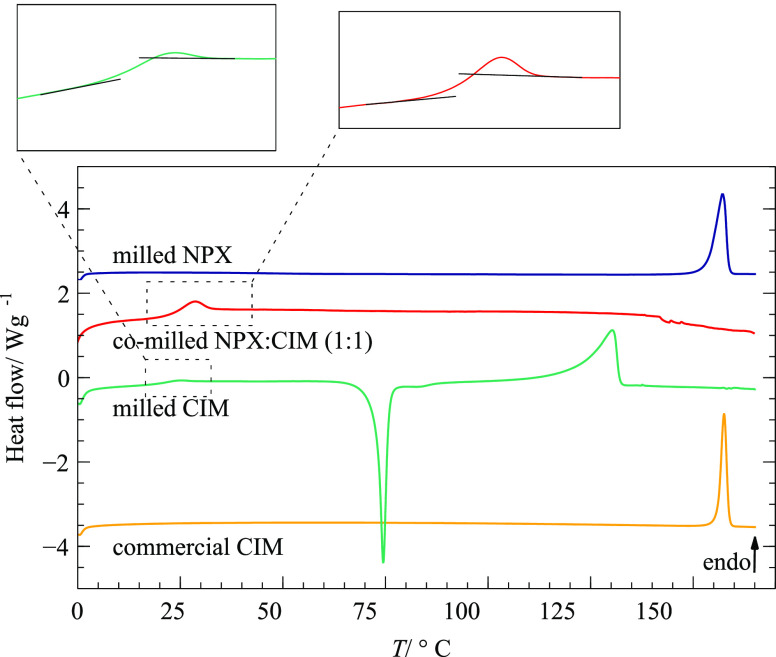
DSC heating thermograms of commercial CIM, milled pure NPX and CIM, and co-milled (1:1) NPX-CIM mixture. β = 10 °C min^–1^. Small endothermic peaks visible on top of the glass transition steps are due to relaxation enthalpy, related to physical aging in the nonequilibrium glassy state.

The DSC thermograms in Figure 4 show the thermal behavior of commercial CIM, milled pure materials, and the co-milled sample (the commercial NPX thermogram remains unchanged after milling). DSC experiments evidence the success of the co-amorphization process of NPX with CIM, where a single glass transition at *T*_g_ = 28.6 °C can be observed, proving miscibility of the amorphous phases.

The DSC curve of the NPX-CIM co-amorphous shows that it remained as a supercooled liquid after devitrification, as no exothermic (crystallization) nor endothermic (fusion) events were observed. This behavior differs from amorphous CIM, which, after devitrification at *T*_g_ = 21.7 °C, shows cold crystallization, followed by melting at a temperature about 10 °C lower than the starting material, therefore indicating the formation of a different polymorphic form.

The thermal behavior of the samples is described in detail in the Supporting Information. These experiments give the instructor the opportunity to introduce several thermodynamic properties of solid-state obtained by DSC experiments, such as melting enthalpy and entropy, and to discuss phase thermodynamic versus kinetic stability.

## Summary

This experiment provides an excellent opportunity to introduce students to the concept of co-amorphization using a green mechanochemical process. The production of amorphous phases by milling demonstrates the usefulness of mechanochemistry as a powerful technology to achieve mechanical activation while avoiding the use of solvents. This experiment integrates a combination of several methods for structural characterization of solid-state materials, such as XRPD, FTIR, and DSC, where students are stimulated to interpret their experimental results. This laboratory activity is also an excellent opportunity to contextualize co-amorphous systems in the landscape of solid forms of active pharmaceutical ingredients and to discuss thermodynamic and kinetic stability of phases.

The proposed pedagogical goals in Table 1 were assessed by the evaluation of the participation of the students in the prelab session and in the lab reports, which include answering a questionnaire focused on these goals.

The study of this co-amorphous system may lead to further investigations or suggest additional related experiments. These could entail the determination and comparison of apparent solubility and/or dissolution rates of the co-amorphous system versus the pure components or explore the influence of the NPX:CIM molar ratio on the *T*_g_.

## Supporting Information Available

Instructor Notes (PDF)
Student Handout Guide (PDF, DOCX)
Experimental Results - Raw Data (ZIP)
