# Supplementary material for: Introduction to Pharmaceutical Co-amorphous Systems Using a Green Co-milling Technique
Source: J Chem Educ. 2023 Mar 23;100(4):1627–32. doi: 10.1021/acs.jchemed.3c00036 (PMC10100544; doi:10.1021/acs.jchemed.3c00036)
Supplement: Supplementary file 1 — ed3c00036_si_001.pdf [file ed3c00036_si_001.pdf]

Supporting information

Introduction to Pharmaceutical Co-amorphous Systems using a Green Co-milling Technique

Joana F. C. Silva,[1] Mário T. S. Rosado,[1*]
Teresa M. R. Maria,[1] Pedro S. Pereira Silva,[2]
Manuela Ramos Silva,[2] M. Ermelinda S. Eusébio[1*]
[1]CQC-IMS, Dep. de Química, Universidade de Coimbra, Rua Larga, 3004-535 Coimbra, Portugal
[2]CFisUC, Dep. de Física, Universidade de Coimbra, Rua Larga, 3000-370, Coimbra, Portugal

*mario.rosado@qui.uc.pt
*quierme@ci.uc.pt

INSTRUCTOR NOTES

Instructor notes

Brief introduction

Most drug candidates in pharmaceutical development exhibit poor water solubility. The use of amorphous forms of poorly water-soluble drugs is becoming one of the most effective approaches to improve their solubility and dissolution rate and thus enhance drug bioavailability. Compared to their crystalline counterparts, amorphous solids have higher energy and lack the long-range order of molecular packing. An emergent approach for stabilizing the amorphous phase is forming co-amorphous systems, avoiding relaxion to crystals. Pharmaceutical co-amorphous are homogeneous amorphous mixtures made of a drug and low-molecular-weight compounds and/or other drug molecules. The intermolecular interactions between the drug and the co-former and/or the effect of mixing are responsible for an increase of stability of the amorphous phases, avoiding crystallization.

Mechanochemistry is a green chemistry alternative method to traditional chemical procedures, since neither organic solvents nor high temperatures are involved, reducing waste production. Mechanochemical methods, like ball milling, have been used to generate stable co-amorphous systems. Its advantages include lower chemical degradation and higher recovery than other preparation methods, like quench cooling, involving high energy consumption and solvent evaporation, adding the use of bulk organic solvents to the energy inefficiency.

The aim of this laboratory experiment is the mechanochemical synthesis and the characterization of the dual-drug (1:1) co-amorphous system made up of (S)-naproxen and cimetidine. The non-steroidal anti-inflammatory drug naproxen can cause side effects that include gastrointestinal disorders that can be ameliorated by combination with cimetidine, commonly used to treat heartburn and peptic ulcer. The formation of the co-amorphous systems is achieved at room temperature by neat grinding, and the sample is characterized by X-ray powder diffraction (XRPD), differential scanning calorimetry (DSC), and Fourier-transform infrared spectroscopy (FTIR). The same experimental techniques are used to characterize the starting pure materials before and after being submitted to the same mechanochemical procedure, verifying eventual solid phase changes induced by grinding. Finally, the results of the three procedures are compared and discussed.

Laboratory session planning

This experiment is designed for advanced graduate students attending the first year of a Master in Chemistry degree. Students must wear protective gloves, lab coat, and eye protection during the whole experiment. Beforehand, they must read the Material Safety Data Sheets (MSDS) of the chemicals they are going to manipulate and adopt the appropriate precautions.

The students were divided into three groups of six students each and work in pairs. Group 1 started the session by using mechanochemistry to prepare the co-amorphous (1:1) naproxen-cimetidine mixture and to grind the commercial sample of cimetidine, running those simultaneously, since the ball mill has two jars. After the milling process, group 1 characterized the co-milled sample and milled cimetidine by XRPD, FTIR and DSC. Meanwhile, the other two groups, group 2 and 3, carried out the characterization of pure starting samples of naproxen (group 2) and cimetidine (group 3), using the same instrumental methods. Afterwards, these groups performed another grinding experiment for the synthesis of the co-amorphous solid, with naproxen in the second milling jar. Each of the three groups was responsible for characterizing the result of this second grinding experiment by one of the techniques used. The results of all the experiments were shared by the three groups and used to elaborate the experimental report.

During the milling processes the students had the opportunity to begin the laboratory report preparation, and to discuss with the instructor the results so far obtained.

The students prepared all the samples and run all the experiments, except XRPD, which were executed with the aid of a lab technician.

This lab experiment can be applied to classes of 6 to 18 students (divided in 3 groups); however, for each group to complete the work, 8 hours divided in two lab sessions are suggested for smaller classes.

In addition to the planned 60-minute pre-lab session already referred in the main manuscript, for students that did not have previous contact with the characterization techniques, these can be the subject of a previous session dealing with their fundamentals and experimental details.

Adaptation to use a single characterization technique

Instructors wishing to adopt one individual characterization technique due to particular interest, limited instrument availability, or time constraints, can easily adapt this lab experiment to explore their specific capabilities, *e.g.*:

The sensitivity of FTIR to the chemical environment can be used to study what functional groups are involved in intermolecular aggregation in the different solid phases, by analyzing the shifts in particular bands observed after amorphization, or to explain how salt formation is responsible for the disappearance of the carbonyl stretching band in co-milled naproxen.

By experimenting with varying mill grinding frequencies and durations, XRPD peak widths can be analyzed to check for its influence on particle size. Where available, variable temperature XRPD can be also used to monitor amorphization and other phase changes, replacing some DSC functionality with near real-time structural analysis.

The glass transition of amorphous materials can be further explored by DSC. It can include the study of the dependence of the T_g with the NPX:CIM molar ratio or the analysis of the endothermic relaxation of the glass for different glass aging times.

Green chemistry principles

The green chemistry principles mentioned in the main article are summarized in Table S1. These provided a better contextualization of green chemistry with the students.

Table S1- The twelve principles of green chemistry.[1]

	The 12 principles of Green Chemistry (GC)
GC 1	Prevention
GC 2	Atom economy
GC 3	Less hazardous chemical syntheses
GC 4	Designing safer chemicals
GC 5	Safer solvents and auxiliaries
GC 6	Design for energy efficiency
GC 7	Use of renewable feedstocks
GC 8	Reduce derivatives
GC 9	Catalysis
GC 10	Design for degradation
GC 11	Real-time analysis for pollution prevention
GC 12	Inherently safer chemistry for accident prevention

Chemicals and Hazards

All the starting materials were commercially available.
(*S*)-naproxen: 98%, CAS number 22204-53-1. Naproxen was obtained from Sigma-Aldrich. H301: Toxic if swallowed. H302: Harmful if swallowed. H315: Causes skin irritation. H319: Causes serious eye irritation. H335: May cause respiratory irritation. H361: Suspected of damaging fertility or the unborn child.
Cimetidine: 99%, CAS number 51481-61-9. Cimetidine was obtained from TCI Chemicals. H318: Causes serious eye damage. H360: May damage fertility or the unborn child. H373: Causes damage to organs through prolonged or repeated exposure.
Ethanol: CAS number 64-17-5. H225: Highly flammable liquid and vapor.
Naproxen and cimetidine powders are inhalation hazards. Ethanol was used to wash experimental tools and glassware used during the experiment.

Instrumentation used

1. Mixer ball mill

For the co-amorphous synthesis, a total of about 100 mg of mixture of naproxen and cimetidine at a (1:1) molar ratio was used. The individual pure drugs were also milled for comparison. A Retsch MM400 mill, Figure S1a), with 10 mL

stainless steel grinding jars and two 7 mm diameter stainless steel balls per jar, Figure S1b), were used to carry out the mechanochemical activation. All grinding experiments were conducted for 60 min at 30 Hz. A view of co-milled sample in the jar is presented in Figure S2.
Some experimental precautions must be observed: the milling jar and the milling balls must be made of the same material; the two milling stations should be always loaded symmetrically, with the two mixing jars carrying approximately the same mass, and properly secured by screw clamps. If the students need to run only one experiment, an empty jar with no balls must be used in the other milling station.

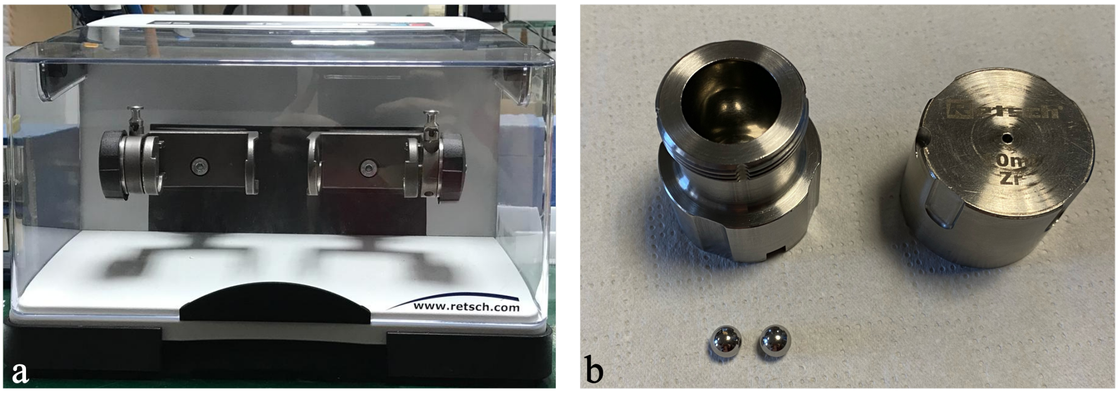

Figure S1- a) Mixer ball mill used in the experiments. b) Empty stainless-steel jar with two stainless steel balls 7 mm diameter.

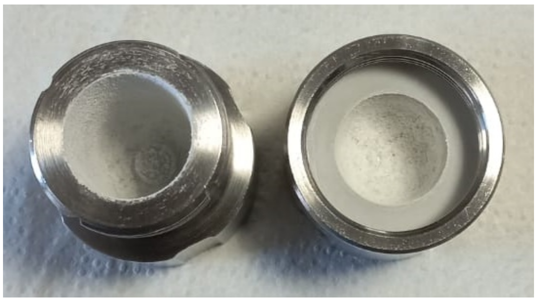

Figure S2- View of the co-milled (1:1) naproxen: cimetidine sample obtained using a mixer mill for 60 min at 30 Hz.

2. X-ray powder diffraction (XRPD)

The diffractograms were obtained with a Bruker D8 Advance diffractometer with Bragg-Brentano reflection geometry, using filtered Cu*K*α (λ = 1.5418 Å) with nickel. The diffractograms were collected in the 2θ range of 4° to 50°, with a step of 0.02° and 0.5s accumulation time per step. A 1D Brucker LINXEYE multidetector was used with energy discrimination for background noise reduction, using 196 pixels corresponding to an adequate acquisition time of 0.5×196 = 96 s per step on a point detector. The incident beam was limited by a divergence slit of 0.3° and Soller slits of 0.5° were also used to limit the divergence of the incident and diffracted beams in the direction perpendicular to the diffraction plane.

3. Differential Scanning Calorimetry (DSC)

DSC thermograms were obtained using a PerkinElmer DSC7 calorimeter with an intracooler cooling unit at −20 °C (ethylene glycol: water, 1:1, V/V, cooling mixture). All measurements were carried out at scanning rate $\beta = 10$ °C min^{-1} under a nitrogen gas flow of 20 mL min^{-1}. Approximately 2 to 5 mg of sample was hermetically sealed in a 30 μL aluminum pan, and a similar empty one was used as reference. Temperature calibration was performed with high grade standards:[2,3] biphenyl (CRM LGC 2610, T_{fus} =(68.93 ± 0.03) °C), and indium (Perkin-Elmer, x = 0.9999, T_{fus} =(156.6) °C). Indium was also used for enthalpy calibration ($\Delta_{fus}H_m$ = 3286 ± 13 J mol^{-1}). Benzoic acid (CRM LGC 2606, T_{fus}= (122.35 ± 0.02) °C) was used to check the calibration. Pyris software for windows, version 3.50 was used.

4. Fourier-transform infrared spectroscopy (FTIR)

Spectra of the solids were recorded at ambient temperature using a Thermo Nicolet IR300 spectrometer with an attached diamond crystal ATR accessory (64 scans, resolution 2 cm^{-1}).

Experimental data

<u>Experimental XRPD data obtained for starting materials, milled and co-milled materials</u>

To compare the initial samples with the milled samples, the students performed X-ray powder diffraction to classify the materials as ordered or disordered solids and determine if the co-milling process was successful.

In Figure S3, the diffractogram of commercial (*S*)-naproxen sample is shown, exhibiting a set of peaks at 6.6°, 12.6°, 13.3°, 16.8°, 18.0°, 19.0°, 20.0°, 20.3°, 22.3°, 22.5°, 23.1°, 23.7°, 24.0°, 25.3°, 27.8°, 28.4°, 29.9°, 31.4°, 32.4°, 33.6°, 34.9°, 35.3°, 38.7°, 39.8°, 40.7° and 45.5°.

Figure S4 shows the X-ray powder diffractogram of (*S*)-naproxen simulated from the CSD entry COYRUD11,[4] which corresponds to our initial sample of pure NPX. Its structure is characterized by hydrogen bonded carboxylic acid chains. Three additional polymorphs of NPX (with different melting temperatures and X-ray diffractograms) were reported by Song and Sohn,[5] prepared from the suspension of the previously described form in three different organic solvents.

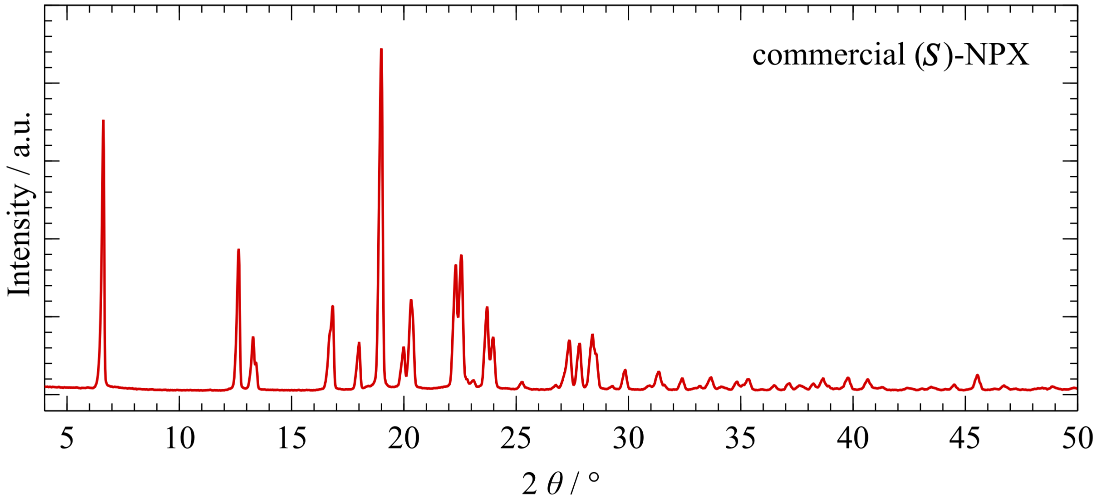

Figure S3- X-ray powder diffractogram of commercial naproxen sample.

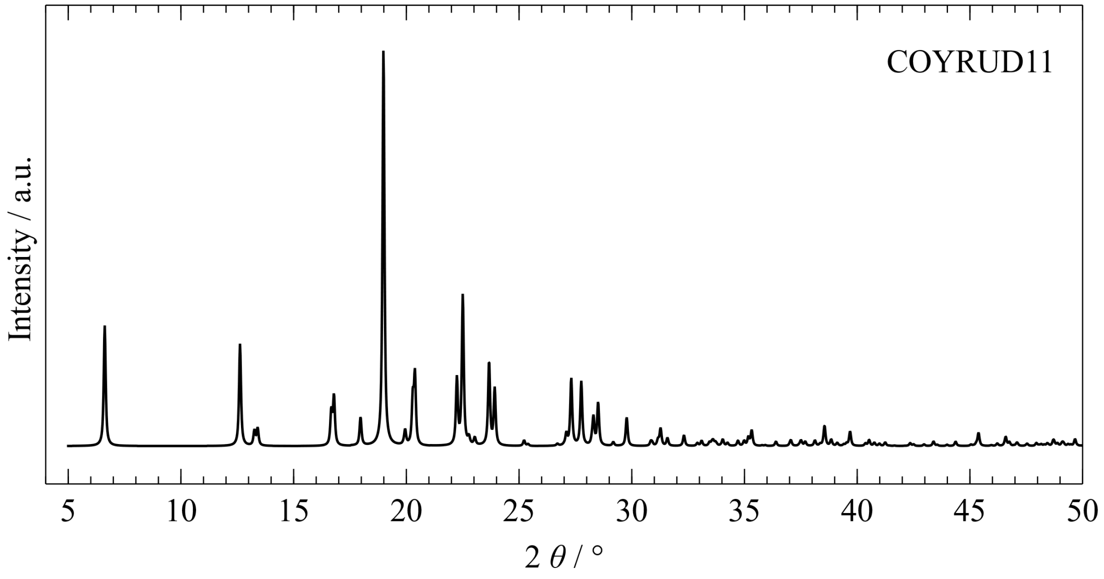

Figure S4- X-ray powder diffractogram of crystalline naproxen, simulated from COYRUD11.[4]

In Figure S5, the diffractogram of milled (*S*)-naproxen is shown, exhibiting a set of peaks at 6.6°, 12.6°, 13.3°, 16.8°, 18.0°, 19.0°, 20.3°, 22.5°, 23.7°, 25.3°, 27.3°, 27.7°, 28.5°, 29.8°, 31.3°, 32.4°, 33.6°, 35.3°, 38.6°, 39.8°, and 45.5°.

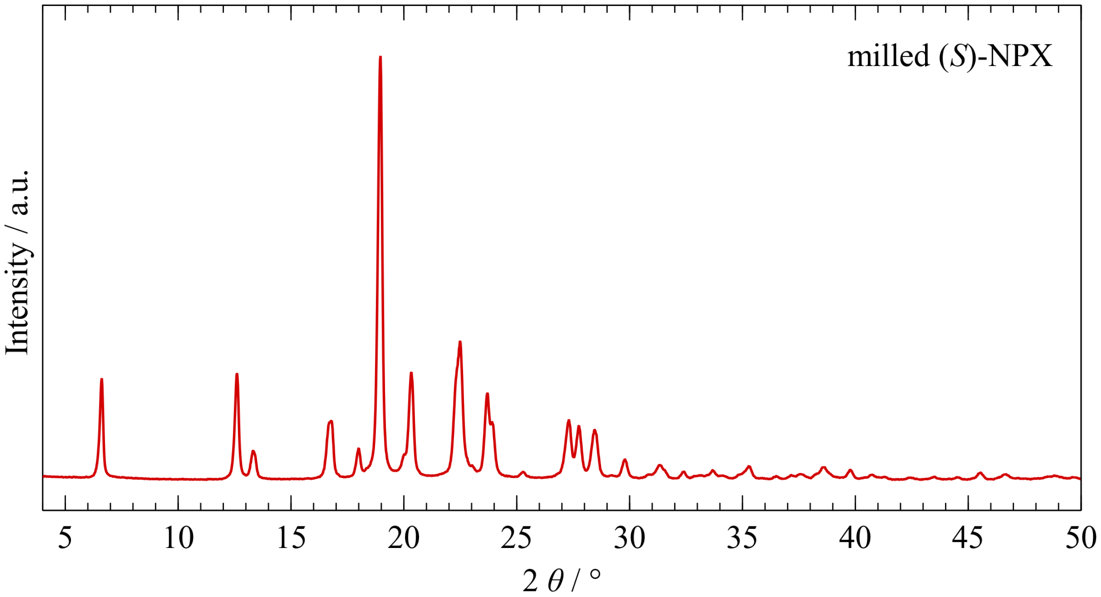

Figure S5- X-ray powder diffractogram of milled naproxen.

In Figure S6, the diffractogram of initial cimetidine sample is shown, exhibiting a set of peaks at 9.4°, 9.6°, 10.0°, 12.9°, 14.7°, 16.5°, 16.5°, 16.8°, 17.8°, 18.4°, 18.8°, 19.0°, 19.6°, 19.8°, 20.5°, 21.2°, 23.2°, 23.5°, 25.3°, 26.0°, 26.8°, 27.2°, 28.0°, 28.4°, 29.1°, 29.9°, 30.4°, 32.6°, 33.7°, 34.5°, 35.0°, 36.0°, 38.6°, 42.5°, 43.2°, 44.6°, 45.7°. Figure S7 shows the simulated X-ray powder diffractogram, simulated from CSD entry CIMETD03.[6] Comparison of diffractograms reveals that they correspond to the same solid form, described by Cernik et al.[6] There are four known polymorphs of CIM (A, B, C and D).[7,8] The starting sample, identified as form A, is characterized by an intramolecular hydrogen bond between the one of the guanidine N-H groups and the -N= atom in the imidazole ring. Form C shows intermolecular hydrogen bonding between imidazole and guanidine moieties, while form D has a characteristic spiral conformation. The structure of form B is yet to be resolved.[8]

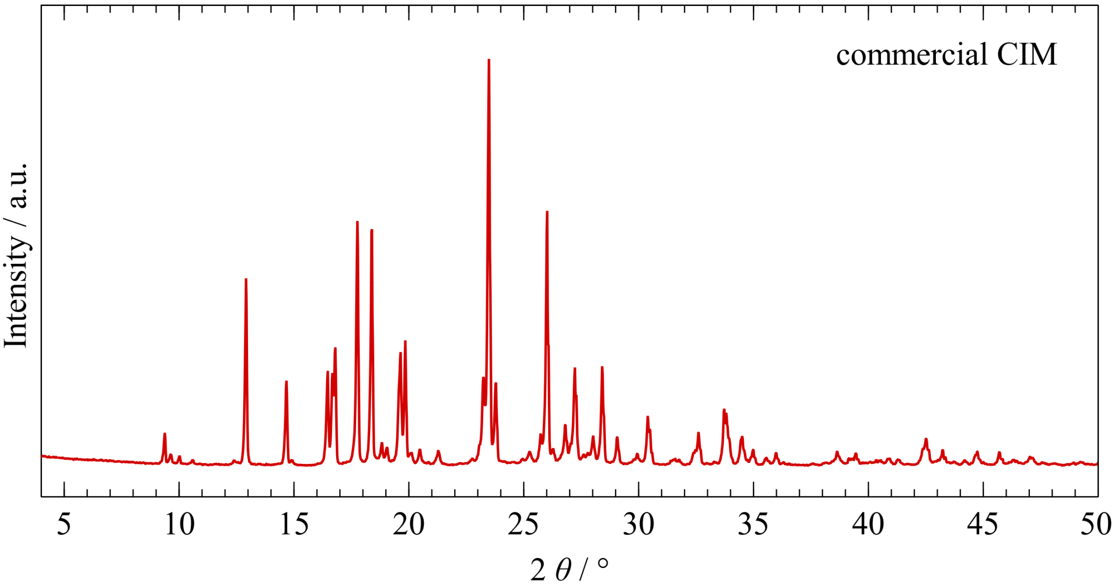

Figure S6- X-ray powder diffractogram of commercial cimetidine.

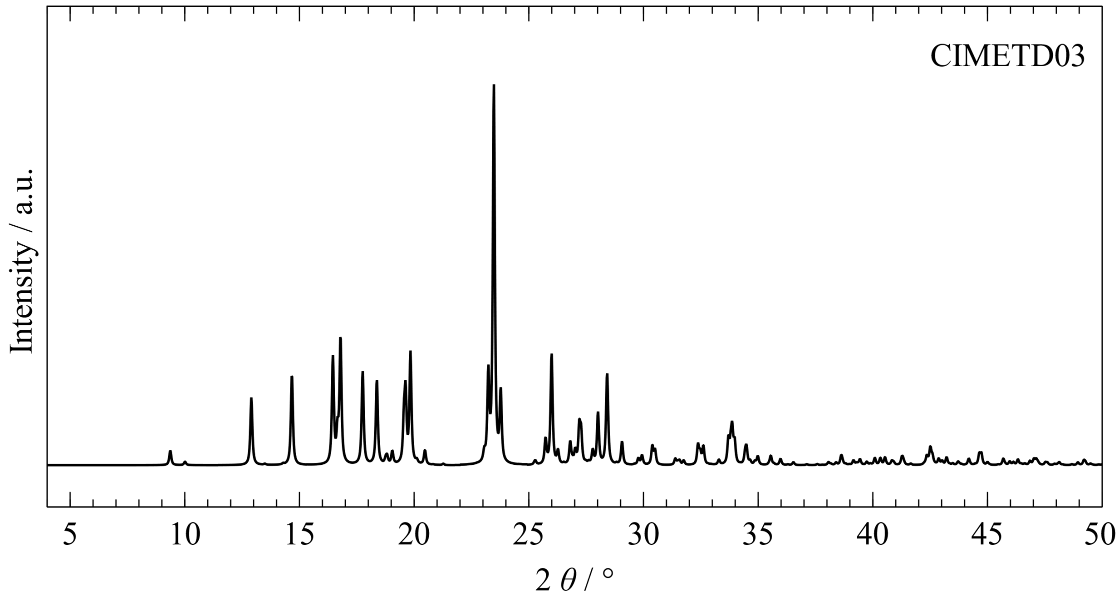

Figure S7- X-ray powder diffractogram of crystalline cimetidine, simulated from CIMETD03.[6]

The diffractogram of milled cimetidine is presented in Figure S8, exhibiting a characteristic halo of an amorphous phase.

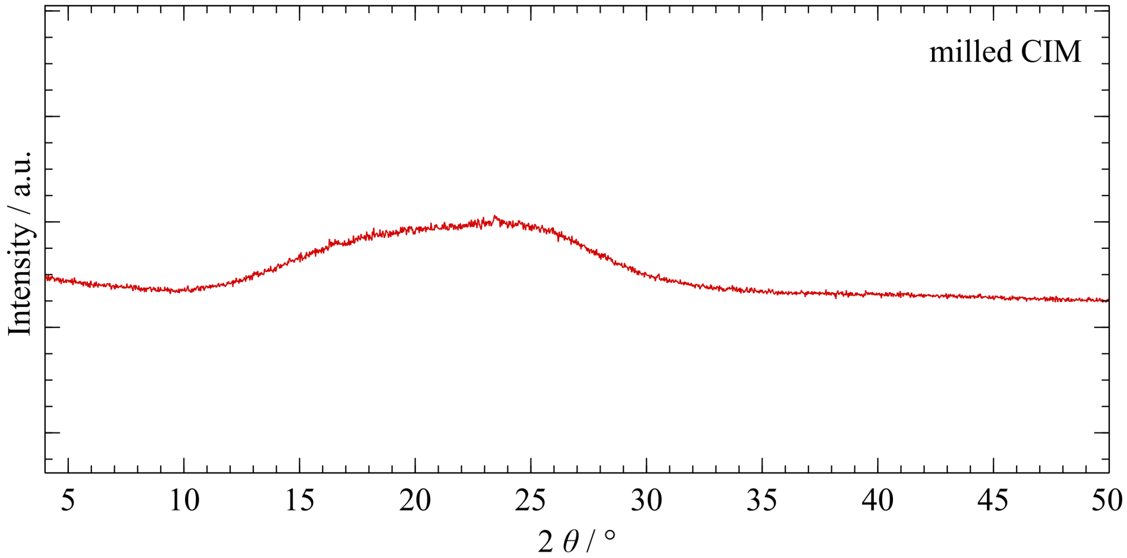

Figure S8- X-ray powder diffractogram of milled cimetidine.

In Figure S9, the amorphous nature of co-amorphous (1:1) naproxen-cimetidine is checked by the of characteristic halo in the X-ray diffractogram, proving that the amorphization process was successful and complete.

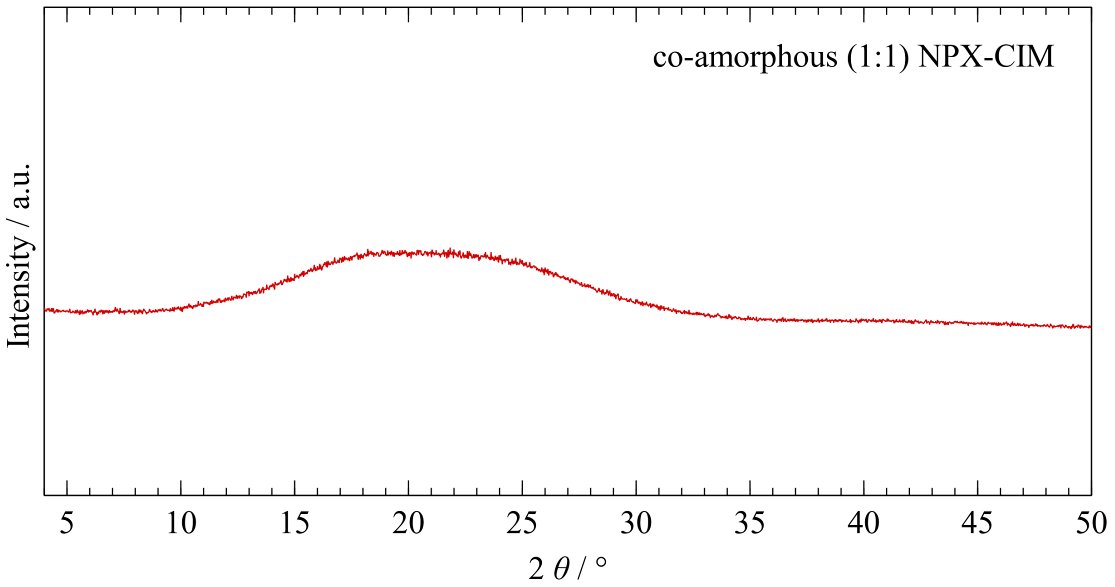

Figure S9- X-ray powder diffractogram of co-amorphous (1:1) naproxen-cimetidine sample.

<u>Experimental FTIR data obtained for starting materials, milled and co-milled materials</u>

The infrared spectrum of the commercial (*S*)-naproxen sample is shown in Figure S10, where the most characteristics bands were marked: the O-H stretching absorption peak with maximum at 3130 cm^{-1}, the aliphatic C-H stretching bands and the carboxylic acid C=O stretching band (1728 cm^{-1}).

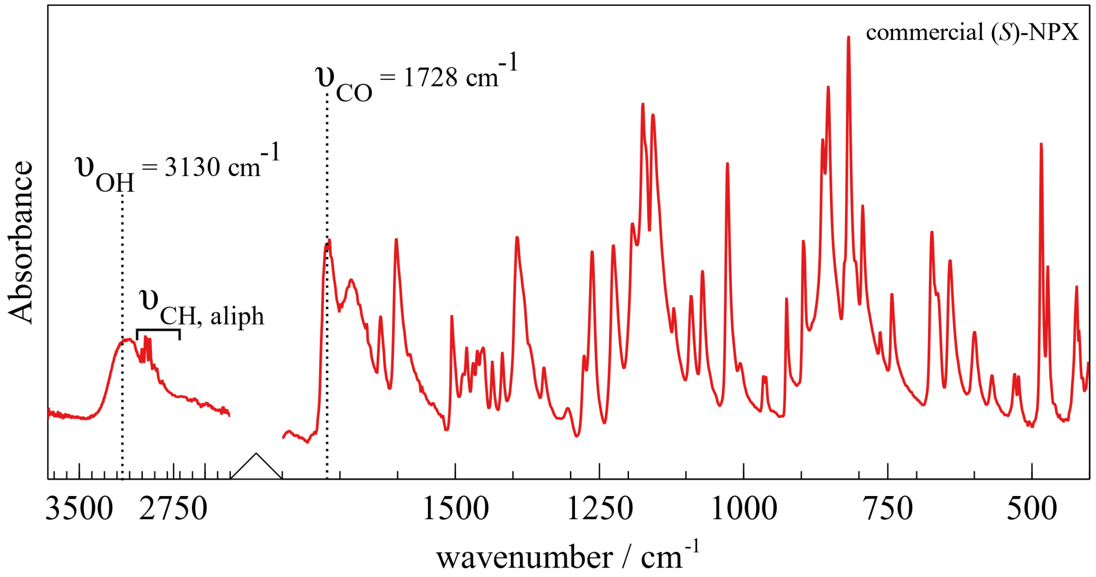

Figure S10- FTIR-ATR spectrum of commercial (*S*)-naproxen sample.

In the FTIR spectrum of milled (*S*)-naproxen, Figure S11, the profile of the absorption bands remains unchanged when compared to the commercial sample, showing that no phase change took place in the milling process, as already demonstrated by the X-ray powder diffraction experiments.

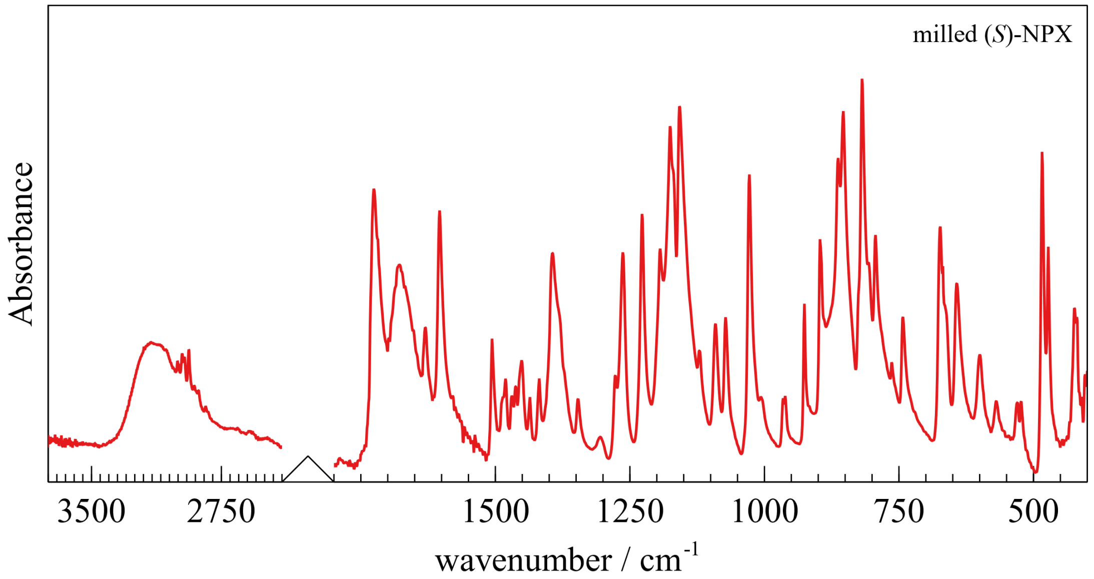

Figure S11- FTIR-ATR spectrum of milled (*S*)-naproxen.

The commercial CIM spectrum, Figure S12, shows a distinct strong absorption at 2172 cm^{-1} corresponding to the nitrile N≡C stretching band. Other marked bands appear at 3320, 3133 and 3036 cm^{-1}, assigned to the N-H stretching vibration, like the aliphatic C-H stretching bands around 2910 cm^{-1}. The strong band at 1584 cm^{-1} corresponds to the symmetric C=C stretching mode.

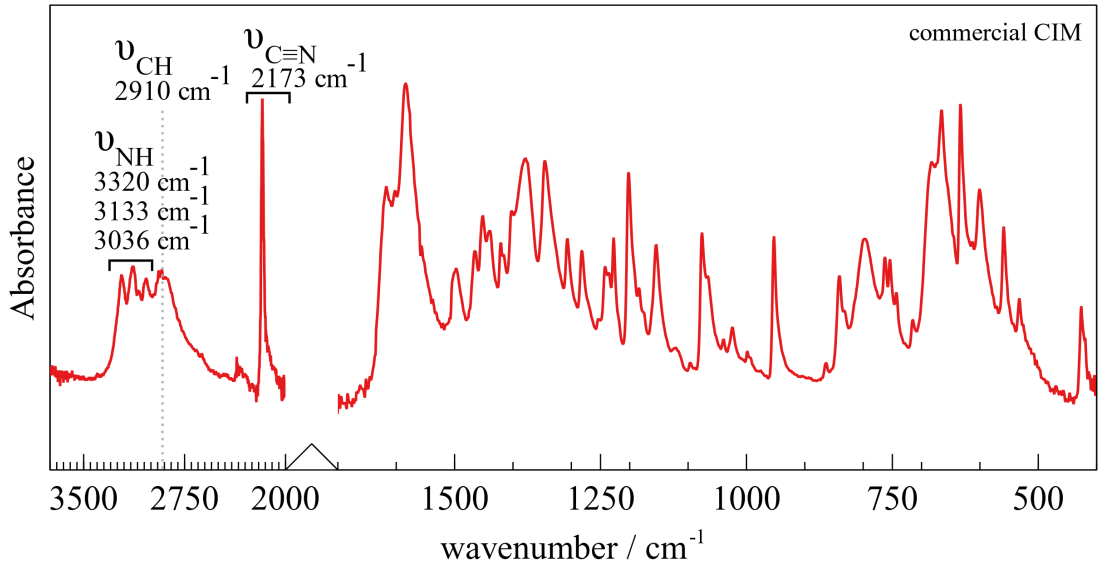

Figure S12- FTIR-ATR spectrum of commercial cimetidine sample.

Cimetidine amorphizes upon milling process, and the resulting IR spectrum, Figure S13, shows marked differences when compared to that of crystalline cimetidine. The N≡C stretching band shifted to 2155 cm^{-1}, and considerable broadening is observed resulting from the lack of long-range order in the bands assigned to both the N-H and the C=C stretching vibrations. Additionally, some other peak shifts are also observed due to changes in the chemical environment. The FTIR spectrum of the co-amorphous mixtures is shown in Figure S14, where the bands referred in the main manuscript are marked.

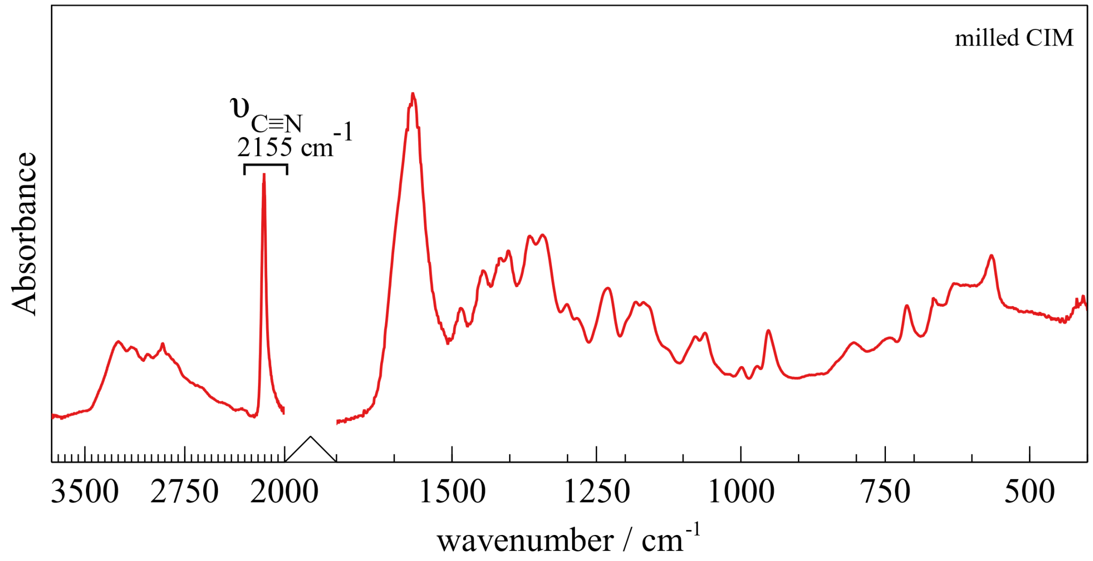

Figure S13- FTIR-ATR spectrum of milled cimetidine sample.

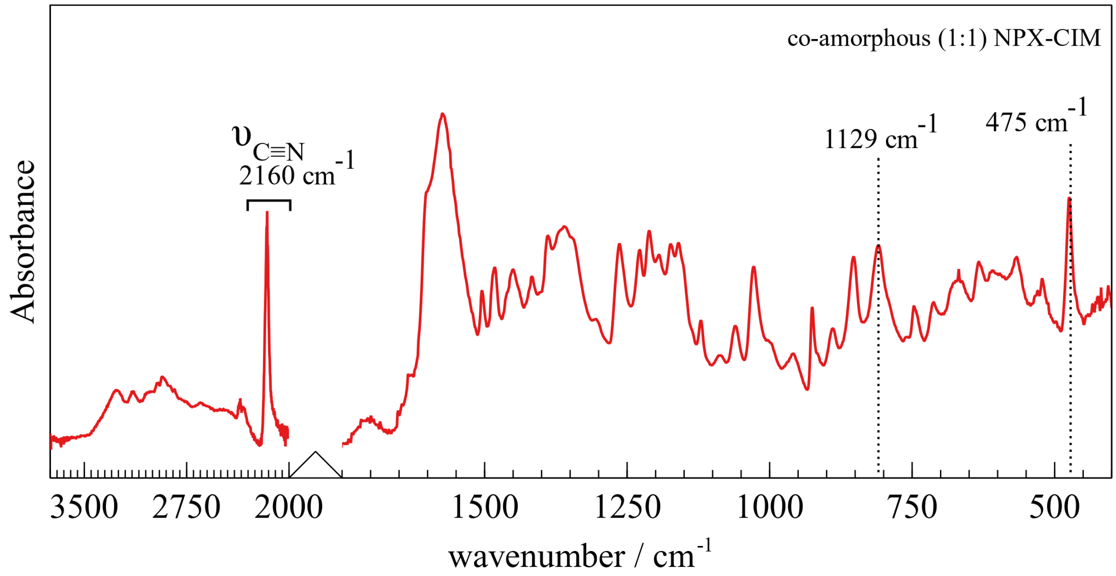

Figure S14- FTIR-ATR spectrum of co-amorphous (1:1) naproxen-cimetidine.

Experimental DSC data obtained for starting materials, milled and co-milled materials

The students performed DSC experiments on the pure initial and pure milled samples and the resulting co-milled mixture.

Figure S15 presents the DSC heating thermograms of a commercial naproxen sample before and after milling. The commercial sample curve shows a single endothermic peak at T = 156.0 °C ($\Delta_{fus}H$ = 32.2 kJ mol^{-1}), corresponding to melting, as does the DSC curve of naproxen after milling, consistent with XRPD results that show that this process did not induce changes in the solid form.

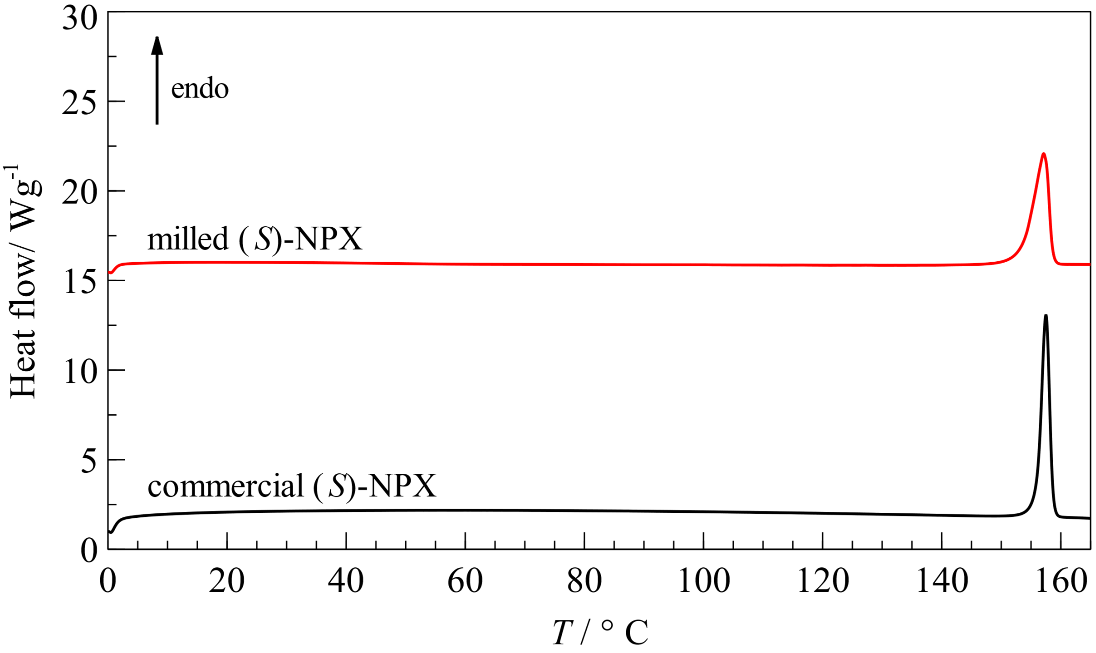

Figure S15- DSC heating thermograms of commercial and milled (*S*)-naproxen; β = 10 °C min^{-1}.

Figure S16 shows the DSC heating profiles of commercial cimetidine and milled cimetidine. The commercial cimetidine curve shows a single endothermic event at T_{fus}= 140.0 °C ($\Delta_{fus}H$ = 41.0 kJ mol^{-1}), corresponding to the melting.

In milled cimetidine, the glass transition is evident at T_g = 21.7 °C, followed by cold crystallization at $T \approx$ 72 °C.

When the supercooled liquid crystallizes, it gives rise to another polymorphic form of cimetidine that melts at T_{fus} = 121.0 °C ($\Delta_{fus}H$ = 26.0 kJ mol^{-1}).

Figure S17 presents de DSC heating curve of the co-milled (1:1) NPX-CIM sample. This co-amorphous system shows a single glass transition event at T_g = 28.6 °C. This finding further confirms the formation of a homogeneous single co-amorphous system. Furthermore, neither melting nor recrystallization peak was observed for the co-amorphous form of NPX-CIM, which is consistent with the full amorphization of the samples indicated by XRPD (Figure S9).

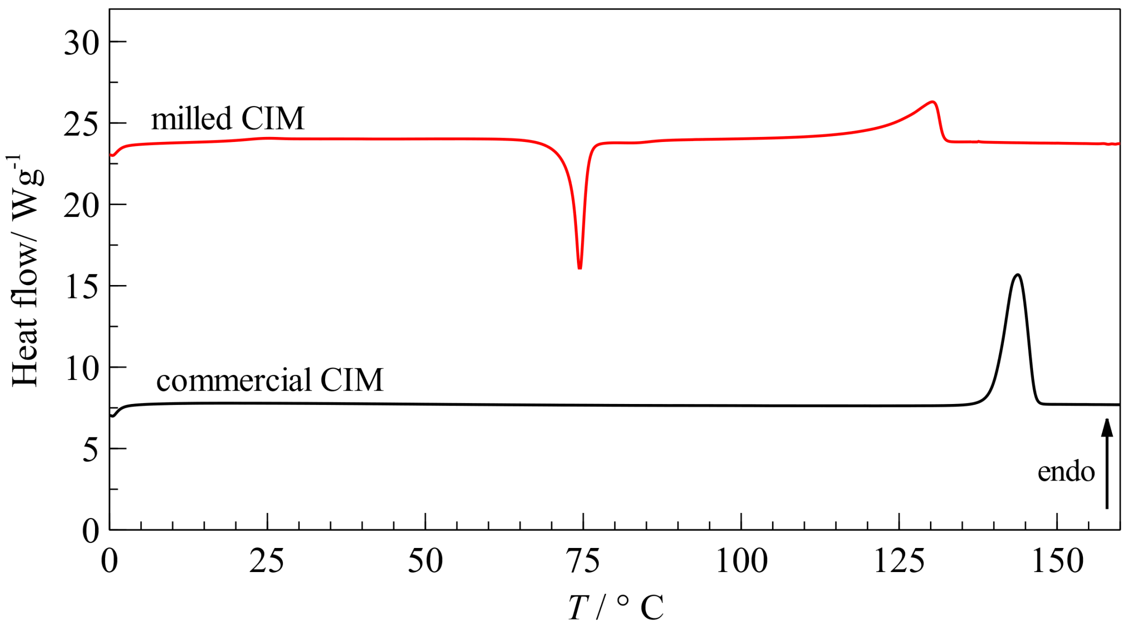

Figure S16- DSC heating thermograms of commercial and milled cimetidine. β = 10 °C min^{-1}.

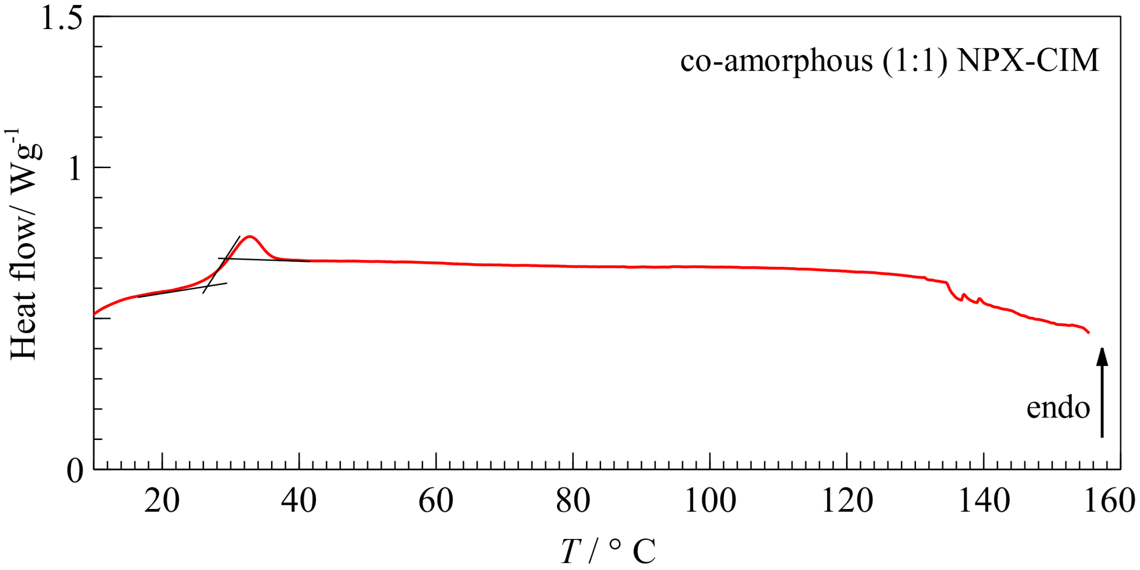

Figure S17- DSC heating thermogram of co-amorphous (1:1) naproxen-cimetidine. β = 10 °C min^{-1}.

Guiding questions for instructors

Guiding questions for instructors about XRPD technique/results:

Q1 Identify the samples where long-range order is present by observation of their X-ray diffraction patterns.

A1 Long-range order is verified by the appearance of sharp peaks characteristic of crystalline phases – commercial NPX, milled NPX and commercial CIM.

Q2 How important is the X-ray powder diffraction technique for the pharmaceutical industry?

A2 X-ray powder diffraction is considered the "gold standard" for the identification and characterization of pharmaceutical solid phases. XRPD is used in the pharmaceutical industry for application in various stages of drug development, manufacturing, and marketing, and establishing and protecting intellectual property because it can be used to identify amorphous materials, lacking diffraction peaks, or to distinguish polymorphs of crystalline API, since crystals with the same chemical composition and different diffraction patterns are polymorphs.

Guiding questions for instructors about FTIR technique/results:

Q1 What is the origin of the bands in FTIR spectra?

A1 The bands in FTIR spectra are originated from transitions between vibrational energy levels, each band corresponding to a vibrational mode. The position of each band is related to bond strengths (force constants) and atomic masses.

Q2 Why is the spectrum of a mixture of solids not always the sum of spectra of pure components?

A2 When the molecular interactions between the different components of a mixture are different from the ones present in the pure solids, some bands can present changes. As each band is usually assigned to a functional group in a molecule, these changes can be used to identify specific molecular interactions that are involved in new supramolecular aggregation like those found in co-amorphous systems. Polymorphism also induces changes in vibrational spectra, which are sensitive to the way that molecules aggregate in condensed phases.

Guiding questions for instructors about DSC technique/results:

Q1 The milled NPX shows a thermal event at 160 °C. Is this event exothermic or endothermic? Discuss what this event at 160 °C represents.

A1 The event at 160°C is endothermic and corresponds to melting of NPX (temperature at which solid and liquid phases are in equilibrium).

Q2 DSC thermograms of commercial CIM and milled CIM after recrystallization show different melting temperatures. What is the meaning of this difference?

A2 The melting temperature is characteristic of a unique solid phase of a pure substance. Different melting temperatures for different solid samples of a pure solid are an indication of polymorphism. Different crystal lattices give rise to changes in the melting point.

Evaluation and scientific report

The laboratory report must be completed in one week after performing the experiment. It should include a brief introduction to co-amorphous drug systems, followed by safety considerations, experimental protocol, and presentation and discussion of the results. This discussion will enable the instructor to assess the successful achievement of the pedagogical goals specified in Table 1 of the manuscript. They can be also assessed by evaluating the answers to a questionnaire included in the end of laboratory report. Several questions and their expected answers are suggested below. The percentages of satisfactory answers obtained by our students are given after each one.

Example of questions which may be raised during the lab session/results

1. List some of the advantages of mechanochemical activation.
   Acceptable answers include better environmental sustainability due to low cost of experiment, minimal or no use of solvents, lack of waste production, high atom economy.
   [72%]

2. What are the main advantages of using co-amorphous drug systems?
   The main advantage of amorphous drug forms is the improved solubility caused by the higher energy. Co-amorphous systems can improve the often-poor stability of amorphous drugs by molecular interactions and segregation with another compound.
   [65%]

3. Give examples of physical changes that can yield exothermic and endothermic peaks in DSC.
   Some solid-solid transitions and melting give rise to endothermic events, while other solid-solid transformations and crystallization originates exothermic events.
   [77%]

4. Describe the differences between crystalline and amorphous solids.
   The fundamental difference between crystalline and amorphous compounds is the order in the arrangement of their constituent units. A crystalline solid has a long-range order, while in contrast, an amorphous solid has only short-range order, with an irregular arrangement of its units. X-ray diffraction of amorphous solids show broad halos, without the peak patterns typical of crystalline materials.
   [90%]

5. Distinguish thermodynamic stability and kinetic stability.
   Thermodynamic stability is related to the permanence in a state because of its lower energy at equilibrium, while kinetical stability is related to the permanence of a system in a higher energy state within a relatively long time-scale because it would need to surmount an energy barrier to change into another. Thus, a system can be kinetically stable, even if it is not at its lowest energy state.
   [50%]

6. Describe glass transition temperature (T_g).
   Glass transition temperature is the temperature at which molecules in the glass phase acquire translational and rotational motions. At the glass transition temperature, the amorphous solids experiment transition from a rigid state to the supercooled liquid.
   [63%]

7. Describe the interactions that could stabilize the co-amorphous preventing them from crystallizing.
   The co-former physically stabilizes the amorphous form either by molecular interactions with the drug (e.g., by salt formation, hydrogen bonding, π-π interactions) or simply by molecular segregation.
   [77%]

8. Give examples of valuable information that can be extracted from infrared spectra of co-amorphous systems.
   The interpretation of the changes in infrared peak frequency can help to understand the changes in molecular interactions in specific functional groups between the pure crystalline API and the co-amorphous system. The broader shape of the bands is an indication of the lesser order in amorphous phases.
   [73%]

9. In what different solid forms can a drug exist?
   Besides the different polymorphs that can exist for a pure crystalline drug, this can be produced as an amorphous solid or in a multicomponent system in combination with co-formers or other API, either crystalline (co-crystals) or amorphous (co-amorphous systems). If it can lead to ion formation, it is a salt. If it incorporates solvent molecule in the crystal structure, it is a solvate.
   [90%]

Our assessment of the answers to the questionnaire in the end of the lab reports and the discussion during the pre-lab session allowed us to conclude that some students were challenged to understand the thermodynamical nature of the glass transition and its manifestation in the DSC thermograms, and the distinction

between thermodynamic and kinetical stability. Some also struggled with the interpretation of the different interactions that could better stabilize co-amorphous systems. Other answers were mostly satisfactory.

Supporting experimental results

For the benefit of instructors implementing this experiment that have limited access to some instrumentation, we are attaching raw text data as CSV files for all the DSC, XRPD and FTIR results as supporting information.

Links to the CSD webpages where all the supporting CIF (crystal information files) can be downloaded:

Naproxen:
https://www.ccdc.cam.ac.uk/structures/Search?Ccdcid=COYRUD11

Cimetidine:
https://www.ccdc.cam.ac.uk/structures/Search?Ccdcid=CIMETD03

The CIF files can be processed by the Mercury software application (available at https://www.ccdc.cam.ac.uk/Community/csd-community/FreeMercury) or other software with similar capabilities to simulate the corresponding X-ray powder diffractograms.

References

(1) Ardila-Fierro, K. J.; Hernández, J. G. Sustainability Assessment of Mechanochemistry by Using the Twelve Principles of Green Chemistry. *ChemSusChem* **2021**, *14* (10), 2145–2162. https://doi.org/10.1002/cssc.202100478.

(2) Gatta, G. D.; Richardson, M. J.; Sarge, S. M.; Stølen, S. Standards, calibration, and guidelines in microcalorimetry. Part 2. Calibration standards for differential scanning calorimetry (IUPAC Technical Report). *Pure and Applied Chemistry* **2006**, *78* (7), 1455–1476. https://doi.org/10.1351/pac200678071455.

(3) Sabbah, R.; Xu-wu, A.; Chickos, J. S.; Leitão, M. L. P.; Roux, M. V.; Torres, L. A. Reference Materials for Calorimetry and Differential Thermal Analysis. *Thermochimica Acta* **1999**, *331* (2), 93–204. https://doi.org/10.1016/S0040-6031(99)00009-X.

(4) Kim, Y. B.; Song, H. J.; Park, I. Y. Refinement of the Structure of Naproxen, (+)-6-Methoxy-α-Methyl-2-Naphthaleneacetic Acid. *Arch. Pharm. Res.* **1987**, *10* (4), 232–238. https://doi.org/10.1007/BF02857746.

(5) Song, J.-S.; Sohn, Y.-T. Crystal Forms of Naproxen. *Arch. Pharm. Res.* **2011**, *34* (1), 87. https://doi.org/10.1007/s12272-011-0110-7.

(6) Cernik, R. J.; Cheetham, A. K.; Prout, C. K.; Watkin, D. J.; Wilkinson, A. P.; Willis, B. T. M. The Structure of Cimetidine (C10H16N6S) Solved from Synchrotron-Radiation X-Ray Powder Diffraction Data. *Journal of Applied Crystallography* **1991**, *24* (3), 222–226. https://doi.org/10.1107/S0021889890013486.

(7) Calvo, N. L.; Maggio, R. M.; Kaufman, T. S. A Dynamic Thermal ATR-FTIR/Chemometric Approach to the Analysis of Polymorphic Interconversions. Cimetidine as a Model Drug. *Journal of Pharmaceutical and Biomedical Analysis* **2014**, *92*, 90–97. https://doi.org/10.1016/j.jpba.2013.12.036.
(8) Shibata, M.; Kokubo, H.; Morimoto, K.; Morisaka, K.; Ishida, T.; Inoue, M. X-Ray Structural Studies and Physicochemical Properties of Cimetidine Polymorphism. *Journal of Pharmaceutical Sciences* **1983**, *72* (12), 1436–1442. https://doi.org/10.1002/jps.2600721217.
(9) Hegedüs, B.; Görög, S. The Polymorphism of Cimetidine. *Journal of Pharmaceutical and Biomedical Analysis* **1985**, *3* (4), 303–313. https://doi.org/10.1016/0731-7085(85)80037-6.
