# Supplementary material for: Introduction to Pharmaceutical Co-amorphous Systems Using a Green Co-milling Technique
Source: J Chem Educ. 2023 Mar 23;100(4):1627–32. doi: 10.1021/acs.jchemed.3c00036 (PMC10100544; doi:10.1021/acs.jchemed.3c00036)
Supplement: Supplementary file 2 — ed3c00036_si_002.pdf [file ed3c00036_si_002.pdf]

*Supporting information*

# Introduction to Pharmaceutical Co-amorphous Systems using a Green Co-milling Technique

Joana F. C. Silva,[1] Mário T. S. Rosado,[1*]
Teresa M. R. Maria,[1] Pedro S. Pereira Silva,[2]
Manuela Ramos Silva,[2] M. Ermelinda S. Eusébio[1*]

[1]CQC-IMS, Dep. de Química, Universidade de Coimbra, Rua Larga, 3004-535 Coimbra, Portugal
[2]CFisUC, Dep. de Física, Universidade de Coimbra, Rua Larga, 3000-370, Coimbra, Portugal

*mario.rosado@qui.uc.pt
*quierme@ci.uc.pt

# STUDENTS HANDOUT GUIDE

# Introduction to pharmaceutical co-amorphous systems using a green co-milling technique

## Introduction

The aqueous solubility of active pharmaceutical ingredients (APIs) is one of the most important parameters for achieving the systemic concentration necessary for the intended biological effect. However, the vast majority of APIs and API candidates exhibit low aqueous solubility, resulting in reduced absorption after oral administration. Thus, one of the major challenges in the pharmaceutical industry is the development of strategies to improve the aqueous solubility of these compounds, thereby incrementing their oral bioavailability. Several techniques are available to improve the aqueous solubility of poorly soluble drugs, including physical modification, such as particle size reduction, or the search for new solid forms (polymorphs, solvates, salts, co-crystals, co-amorphous phases).

The development of amorphous solid pharmaceuticals has been shown to be an effective approach to increase the aqueous solubility of APIs. Amorphous solids differ from crystalline materials by the absence of long-range structural order, keeping only short-range order. On thermodynamic grounds, amorphous solids are unstable or metastable, being in a higher energy state relatively to the crystalline form. Therefore, they may exhibit low physical stability due to their tendency to relax, crystallizing after some time. Thus, to circumvent the low stability inherent to the amorphous state, several techniques have been proposed for kinetic stabilization of amorphous compounds, namely the formation of co-amorphous materials. These are multicomponent amorphous solids that result from the combination of an API with a pharmaceutically acceptable low molecular weight co-former, or with another API in a dual-drug perspective. Therefore, co-amorphous formulations aim at increasing, not only the aqueous solubility, but also the physical stability of the disordered solid. The increased stability of co-amorphous solids is due to mixing effects and, mainly, to the establishment of intermolecular interactions between the drug and the co-former.

Among the various techniques available for preparing co-amorphous systems (rapid cooling of melts, rapid solvent evaporation from a solution of the two components, freeze-drying...), mechanochemistry has proven to be an effective technique, a green alternative to classical processes: no organic solvents or high temperatures are required, also reducing the amount of chemical waste. Consequently, mechanochemical processes have some advantages that include lower chemical degradation and higher recovery than other methods of amorphous preparation, which may involve high energy consumption and/or high consumption of organic solvents.

The biopharmaceutical classification system, BCS, groups APIs into four groups according to their aqueous solubility and permeability. According to this classification, class II compounds have low solubility and high permeability and class IV compounds present low solubility and low permeability. These APIs are preferential targets for co-amorphous phase investigation.

## Objectives

- To produce, using green mechanochemical methods, an equimolar co-amorphous binary system of cimetidine and naproxen, Figure S1, both classified as BCS class II.

- To characterize the starting solids and those submitted to the milling process, by several physicochemical techniques: differential scanning calorimetry, infrared spectroscopy and X-ray powder diffraction.

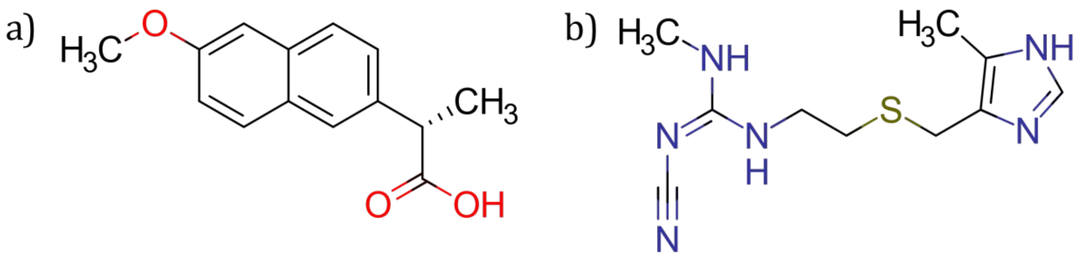


Figure S1- Chemical structure of (a) naproxen, and (b) cimetidine.

## Materials and safety warnings

***(S)-naproxen: 98%, CAS number 22204-53-1***: H301: Toxic if swallowed. H302: Harmful if swallowed. H315: Causes skin irritation. H319: Causes serious eye irritation. H335: May cause respiratory irritation. H361: May damage fertility or the unborn child.

***Cimetidine: 99%, CAS number 51481-61-9***: H318: Causes serious eye damage. H360: May damage fertility or the unborn child. H373: May cause damage to organs through prolonged or repeated exposure.

## General procedure

**Mechanochemistry –** To synthesize the co-amorphous system, weigh a total of 100 mg cimetidine and (*S*)-naproxen in an equimolar ratio. Grind the mixture during 60 min at 30 Hz in the oscillatory ball-mill. The individual components should be submitted to the same process to account for the effects of grinding in the solid phase. Notice that the milling jar and the milling balls must be made of the same material; the two milling stations should be always loaded symmetrically, with the two mixing jars carrying approximately the same mass, and properly secured by

screw clamps. If you need to run only one experiment, an empty jar with no balls must be used in the other milling station. Take special care to not heat the jars in your hands and avoid water vapor condensation on the materials that can lower the glass transition temperature.

**Differential Scanning Calorimetry** – These experiments are useful to characterize the thermal behavior of the samples, including the melting temperature for crystalline solids and the glass transition for amorphous materials. Prepare DSC pans and submit them to a heating run from 0 to 165 °C at 10 °C/min. Repeat the procedure for all starting materials and ground solids.

**Infrared Spectroscopy (ATR-FTIR) and X-Ray Powder Diffraction** – Collect infrared spectra and powder diffractograms for all samples.

## Suggested reading


Allesø, M.; Chieng, N.; Rehder, S.; Rantanen, J.; Rades, T.; Aaltonen, J. Enhanced Dissolution Rate and Synchronized Release of Drugs in Binary Systems through Formulation: Amorphous Naproxen–Cimetidine Mixtures Prepared by Mechanical Activation. *Journal of Controlled Release* **2009**, *136* (1), 45–53. https://doi.org/10.1016/j.jconrel.2009.01.027.

Shi, Q.; Moinuddin, S. M.; Cai, T. Advances in Coamorphous Drug Delivery Systems. *Acta Pharmaceutica Sinica B* **2019**, *9* (1), 19–35. https://doi.org/10.1016/j.apsb.2018.08.002.

Delori, A.; Friščić, T.; Jones, W. The Role of Mechanochemistry and Supramolecular Design in the Development of Pharmaceutical Materials. *CrystEngComm* **2012**, *14* (7), 2350–2362. https://doi.org/10.1039/C2CE06582G.

Do, J.-L.; Friščić, T. Mechanochemistry: A Force of Synthesis. *ACS Cent. Sci.* **2017**, *3* (1), 13–19. https://doi.org/10.1021/acscentsci.6b00277.
